# Dynamic Ultrasound Evaluation Supraspinatus Tendon Injuries Using a Pilates Elastic Band

**DOI:** 10.1002/atn2.70066

**Published:** 2026-05-22

**Authors:** Viet‐Tam Van, Charlotte Vifquain, Romain Nicolle, Nicolas Sans, Nicolas Bonnevialle, Marie Faruch‐Bilfeld

**Affiliations:** ^1^ Département de Radiologie et Imagerie Médicale CHU Toulouse Toulouse France; ^2^ Département de Chirurgie orthopédique et traumatologique CHU Toulouse Toulouse France

## Abstract

Supraspinatus tendon tears are a common cause of shoulder pain and dysfunction. Although magnetic resonance imaging and computed tomography arthrography are still considered the gold standard, ultrasound offers a reliable, accessible, and radiation‐free alternative. However, identifying tears can be challenging, particularly for less experienced operators. This technical note describes a simple dynamic ultrasound maneuver employing a Pilates elastic band. With the patient in a modified Crass position, the band is secured around the pelvis to provide controlled resistance during active lateral elevation of the shoulder. This loading mobilizes joint fluid and applies physiological stress to the tendon, thereby enhancing visualization of tears through gapping or altered gliding. This simple, well‐tolerated technique could improve diagnostic accuracy, as well as guiding rehabilitation and surgical decisions.

## Graphical abstract

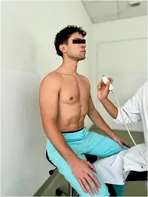

**VIDEO 1:** Dynamic Maneuver. This video shows the patient setup, probe positioning, and dynamic ultrasound assessment of the supraspinatus tendon under controlled resistance using a Pilates resistance band. Step 1—Initial position: The patient is seated with the hand placed on the lower back (modified Crass position) and the elbow flexed at 90°. The transducer is positioned over the supraspinatus tendon in the oblique coronal plane. This position reproduces the standard approach used for rotator cuff evaluation and ensures reproducibility. Step 2—Dynamic maneuver (with Pilates resistance band): The same setup is maintained while a Pilates resistance band is attached around the pelvis to apply gentle counter‐resistance during active lateral elevation of the arm. The patient slides the hand into the elastic band, palm facing the lower back with the thumb placed outside the band. The purple arrow indicates the importance of keeping the elbow aligned with the trunk, maintaining a strictly lateral elevation plane to avoid compensatory forward movement. The blue angular arrow represents the direction of active and external elevation and the axis of movement under resistance, emphasizing controlled and reproducible tendon loading. Step 3—Dynamic ultrasound findings: The supraspinatus tendon is examined dynamically during active elevation. The green arrow identifies the contracting deltoid fibers, confirming correct activation and alignment. The pink arrow highlights the hypoechoic gap corresponding to the supraspinatus tendon tear. The yellow stars indicate the proximal and distal tendon stumps, separated by the fluid‐filled defect revealed during the resisted maneuver, confirming a transfixing tear. Step 4—Comparative assessment (standard vs Pilates method): Side‐by‐side ultrasound images show the same supraspinatus tendon without (left) and with (right) dynamic resistance. Under standard conditions, the tear may appear subtle or incomplete. When resistance is applied using the Pilates band, the separation of the tendon stumps and the presence of fluid within the defect make the transfixing lesion clearly visible, even for less experienced operators. Video content can be viewed at https://doi.org/10.1002/atn2.70066.

Supraspinatus tendon injuries are a leading cause of shoulder pain and functional impairment, with a high prevalence in the general population.^1^ ^,^ ^2^ Although magnetic resonance imaging (MRI) and computed tomography arthrography remain the reference standards for tendon assessment, recent advances in musculoskeletal ultrasound (US) have positioned it as a reliable alternative.

A recent meta‐analysis published by Farooqi et al. even shows statistically equivalent capability to magnetic resonance imaging in the diagnosis of both full‐ and partial‐thickness rotator cuff tears, with a sensitivity of over 83%.^3^

Thanks to its accessibility, cost‐effectiveness, radiation‐free nature, and reliable diagnostic performance, US has become a vital imaging tool for evaluating the shoulder. Nevertheless, carrying out a comprehensive shoulder US check is still technically challenging, particularly for radiologists lacking speciality status or experienced in the early stages of their career.

The purpose of this technical note is to describe a dynamic US method using a Pilates band to improve supraspinatus tendon appreciation and facilitate lesion detection.

## TECHNIQUE

**FIGURE 1:**
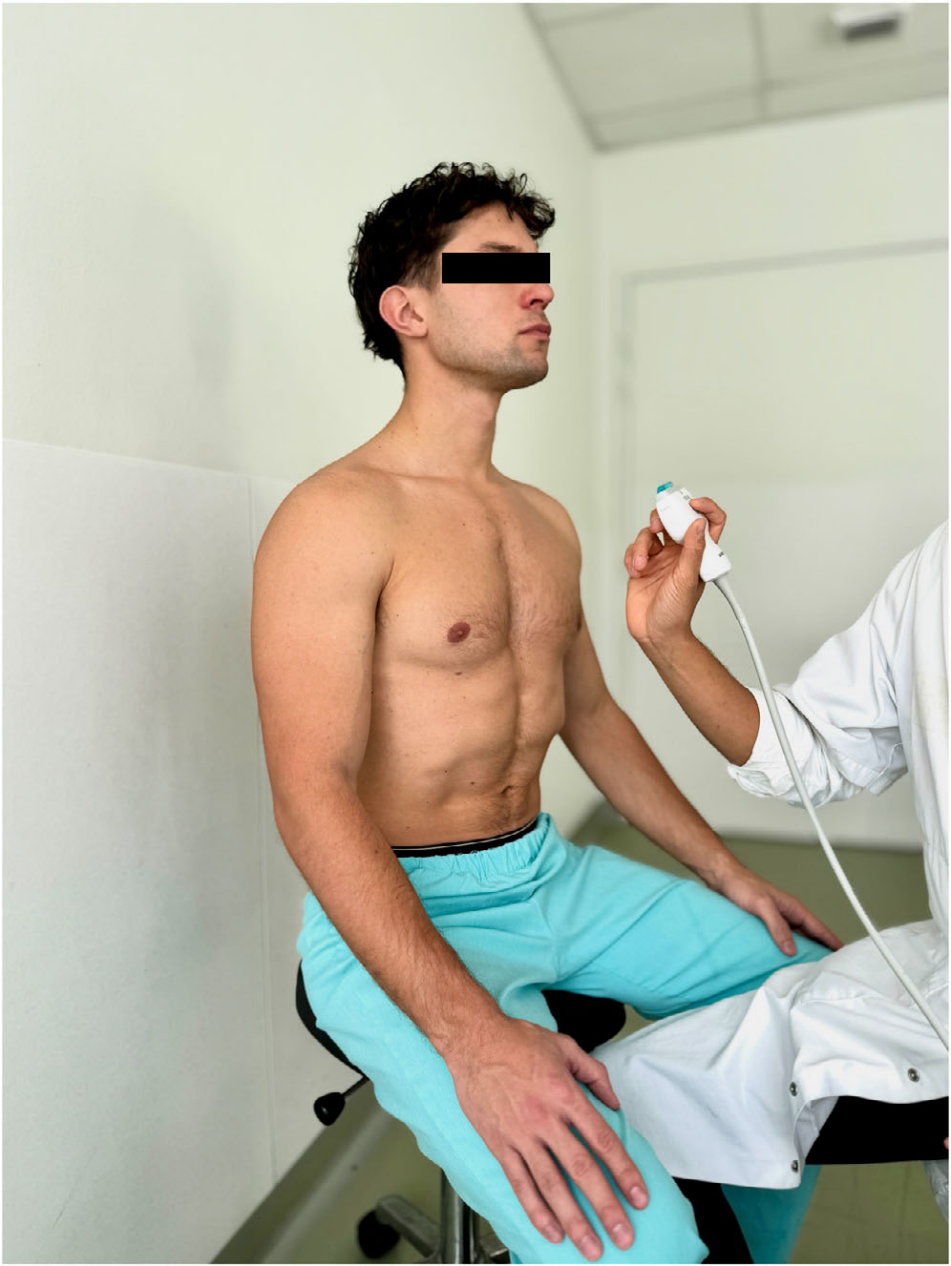
Initial position. For the initial shoulder ultrasound setup, the patient is seated while the examiner positions themselves very close, almost in a staggered alignment, placing 1 knee between the patient's 2 knees. The patient stays in a relaxed position with the back straight and both arms resting naturally. A linear ultrasound transducer is used for the examination. This setup provides a stable, comfortable position for both the patient and the examiner, facilitating accurate and reproducible evaluation of the supraspinatus tendon.

**FIGURE 2:**
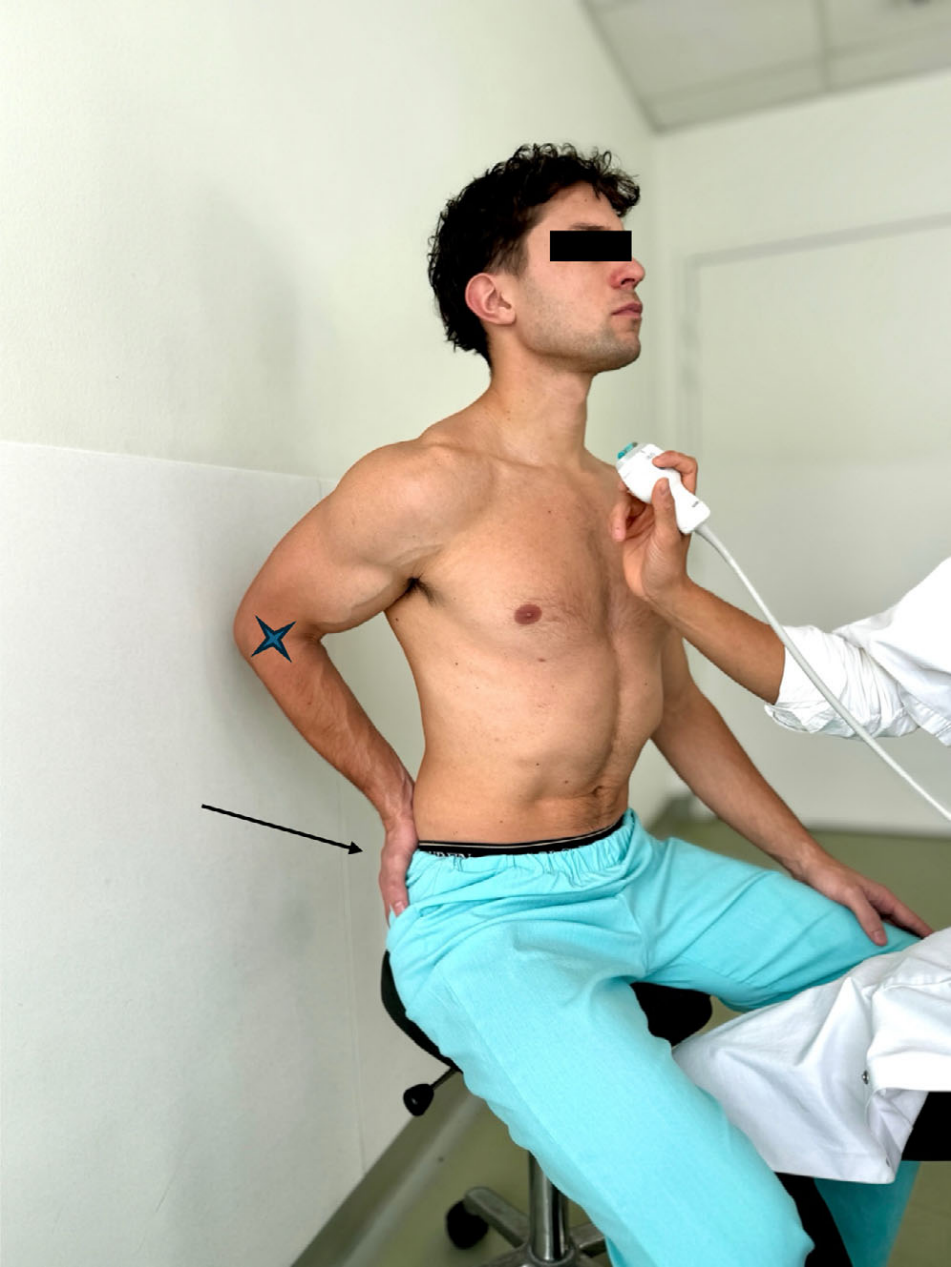
Standard modified Crass position. The patient places the hand on the lower back (arrow), elbow flexed at 90° and aligned with the trunck (blue star), in a modified Crass position. This posture brings the supraspinatus tendon forward and allows tensioning of the muscle fibers. This is the standard position for static evaluation of the supraspinatus tendon.

**FIGURE 3:**
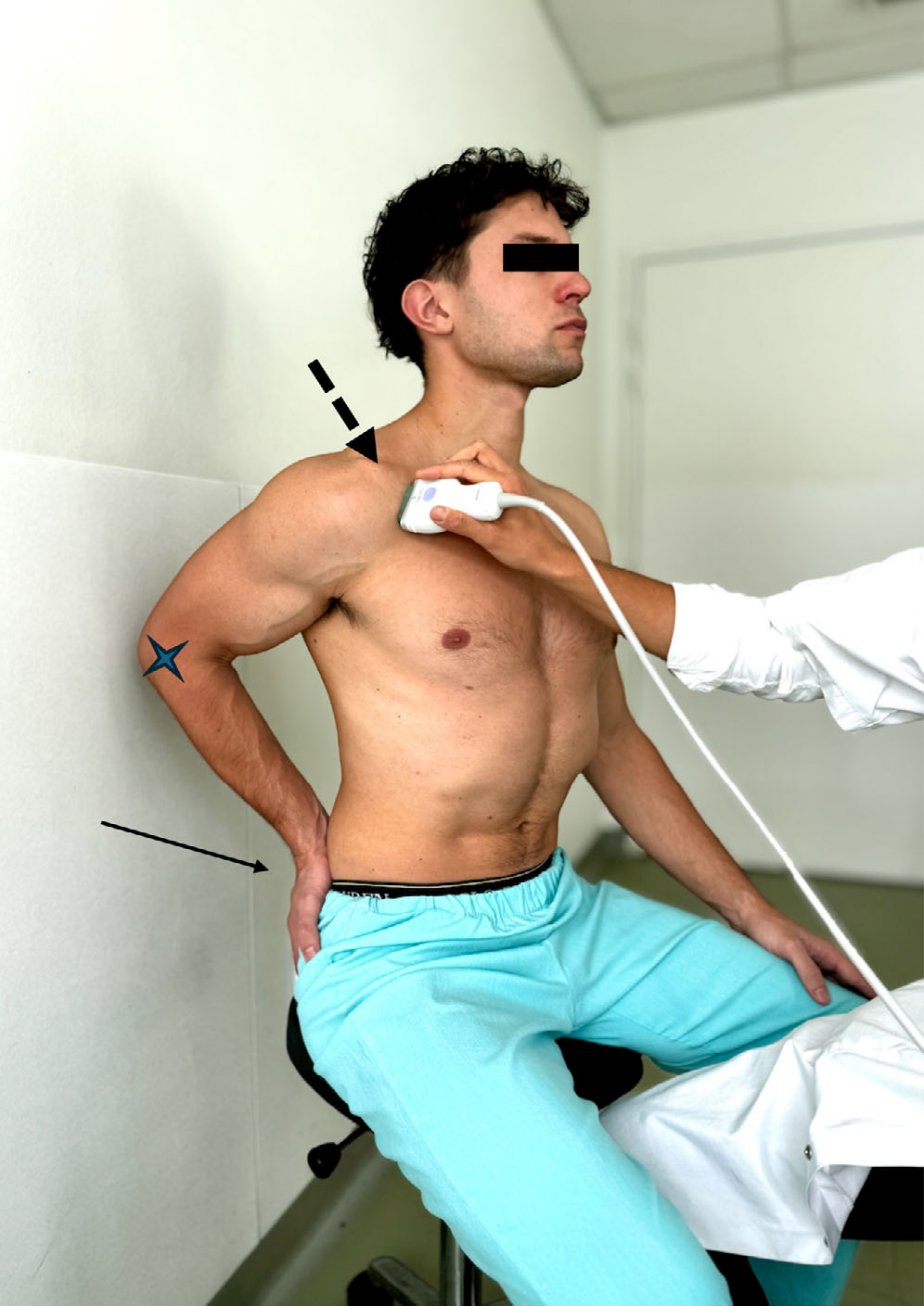
Ultrasound probe position. The patient places the hand on the lower back (arrow), elbow flexed at 90° and aligned with the trunck (blue star), in a modified Crass position. The probe is positioned in the deltopectoral groove to visualize the tendon along its full axis (dashed black arrow) in a coronal oblique plane.

A high‐frequency linear probe (18‐24 MHz) (Aplio 500, Toshiba, Tokyo, Japan) is used to perform the examination (Figures 1, 2, 3). During the dynamic evaluation of the supraspinatus tendon, a Pilates resistance band is used to provide controlled isometric resistance.

**FIGURE 4:**
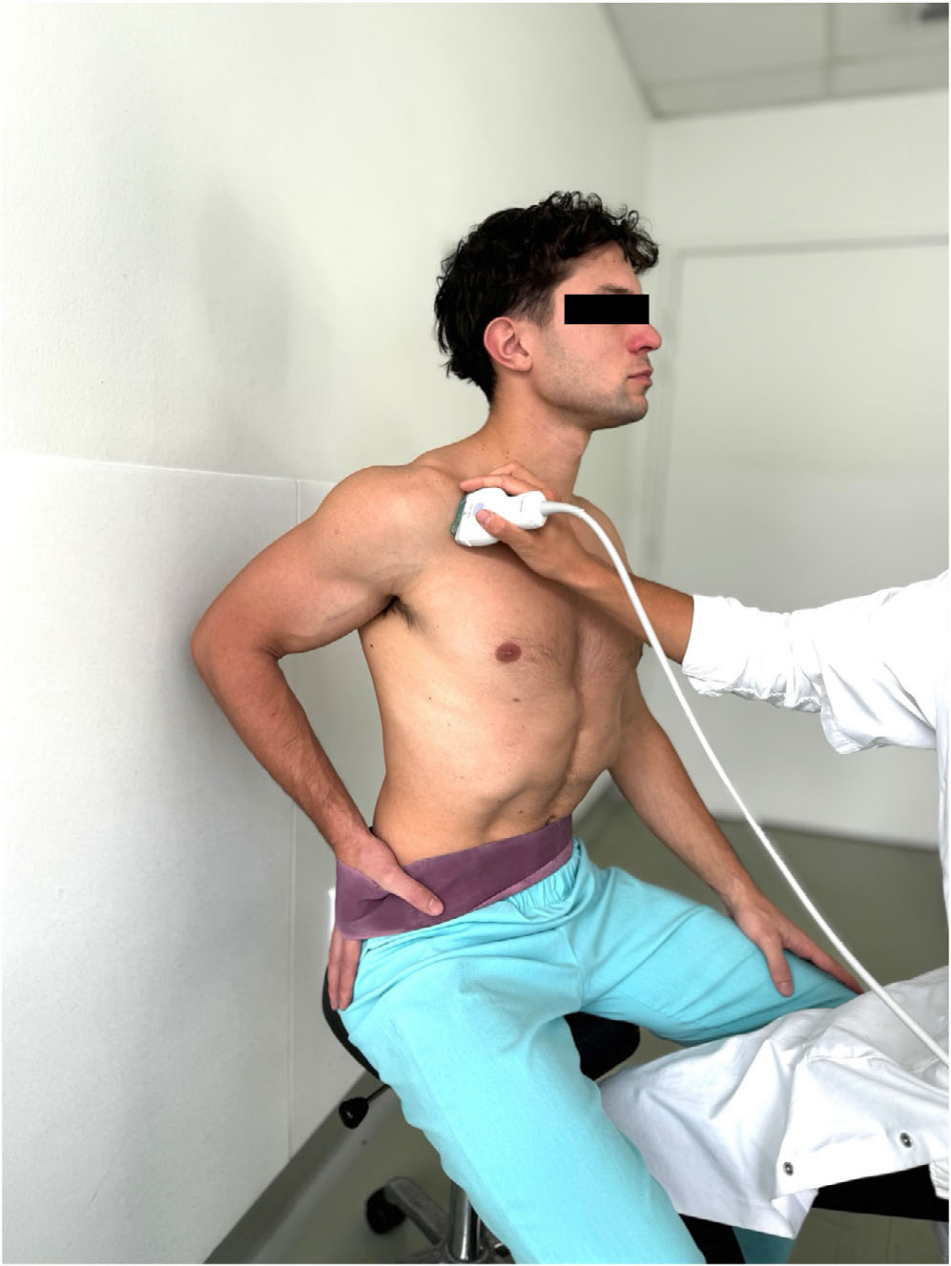
Based position for dynamic exploration with Pilates elastic band. For dynamic exploration under controlled resistance, the patient is positioned identically to the standard method. A Pilates elastic band (purple arrow) is firmly secured around the trunk to provide uniform counter‐resistance during movement. The patient slides their hand under the band with their thumb placed externally (red star), which maintains proper alignment and prevents compensatory movement of the wrist or elbow. Ultrasound probe is oriented in the coronal oblique plane, in the deltopectoral groove (dashed black arrow).

The patient is seated with the hand placed on the lower back (in a modified Crass position) and the elbow flexed at 90° (Figures 2 and 3). The Pilates band is attached around the subject's pelvis and fixed in place to counteract the lateral elevation of the shoulder (Figure 4).

**FIGURE 5:**
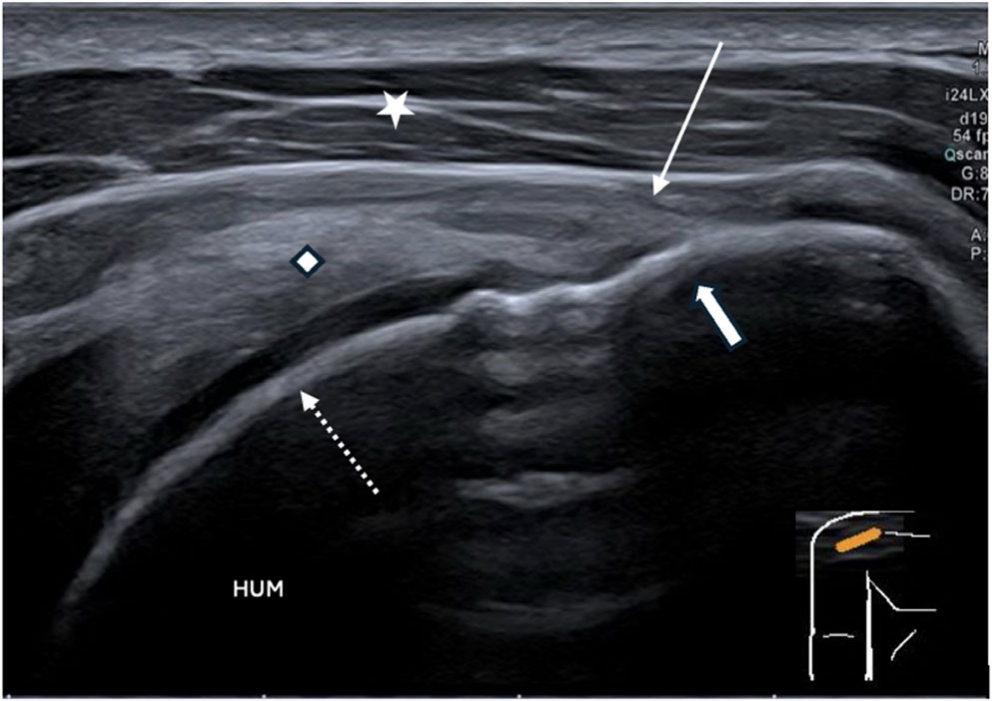
Coronal oblique ultrasound view of the right supraspinatus tendon, obtained with the patient positioned according to the setup illustrated in Figure 3, using the standard ultrasonographic technique. Coronal oblique (long‐axis) ultrasound image of the supraspinatus tendon obtained with the patient in the *modified Crass position*. The probe is positioned in the deltopectoral groove to visualize the tendon along its full axis. The white star denotes the deltoid muscle, and the white diamond indicates the supraspinatus tendon. The thick white arrow shows the footprint at the humeral head (HUM), while the dotted arrow outlines the humeral cartilage. The thin white arrow highlights focal flattening at the distal tendon insertion, with increased echogenicity and fiber distortion suggesting a tear.

**FIGURE 6:**
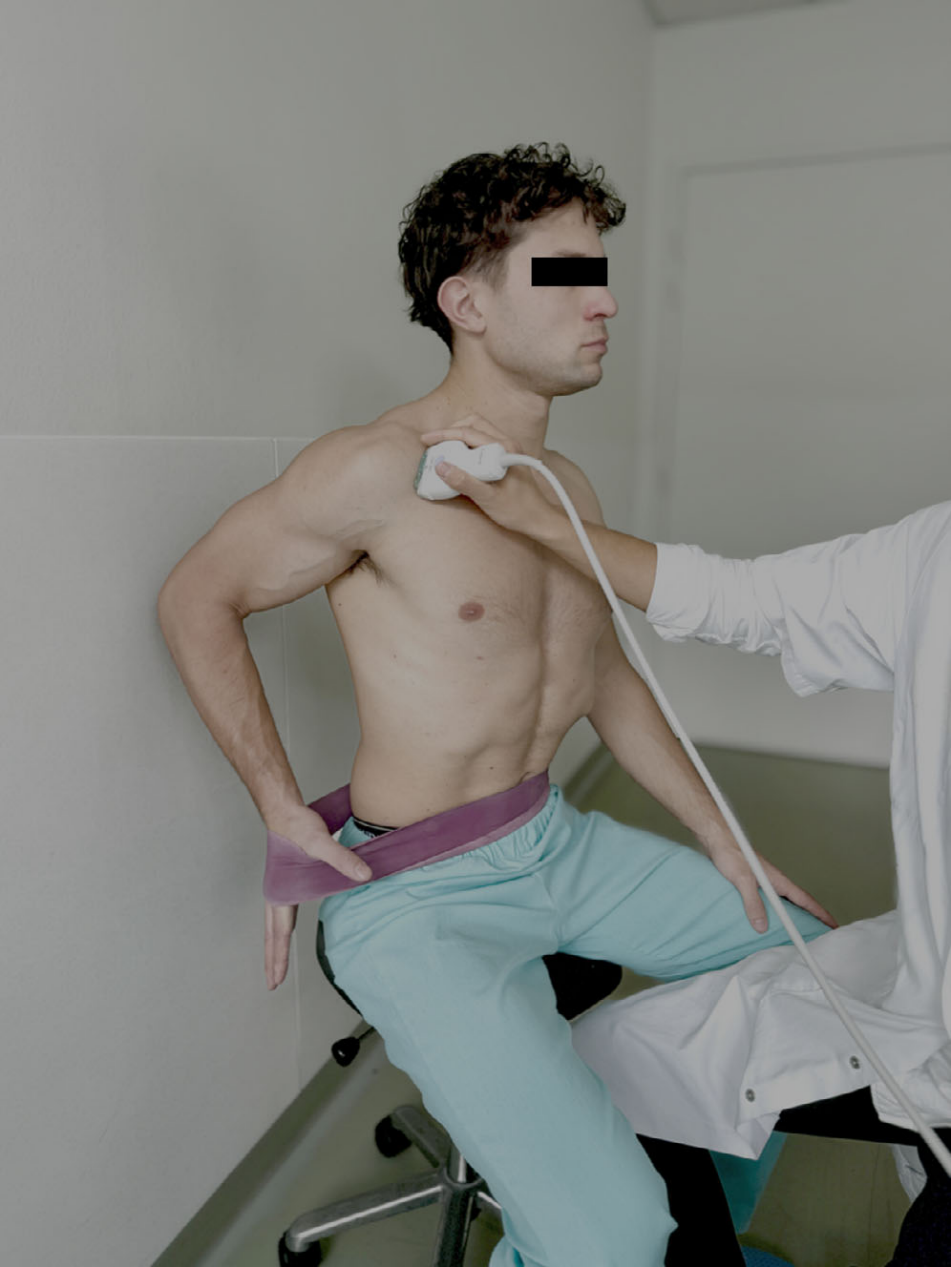
Dynamic phase movement. During dynamic phase, the patient performs active lateral elevation of the shoulder against the Pilates elastic band (blue arrowhead). The blue star shows the correct alignment of the elbow with the trunk. This alignment must be maintained throughout the maneuver to avoid compensatory forward motion. The probe is kept in a stable position over the supraspinatus tendon throughout the maneuver to allow continuous visualization of tendon motion (dashed black arrow).

**FIGURE 7:**
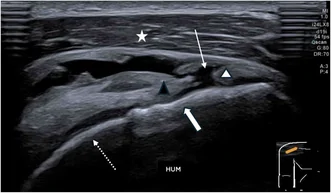
Coronal oblique ultrasound view of the right supraspinatus tendon, obtained with the patient positioned according to the setup illustrated in Figure 6, using the Pilates resistance band method. Coronal oblique (long‐axis) ultrasound image obtained while the patient performs active lateral elevation of the shoulder against a Pilates resistance band. The white star shows the proper alignment of the *deltoid muscle* fibers, which is a key indicator of correct patient positioning. The thin arrow points to the *tear of the supraspinatus tendon unmasked by the dynamic maneuver.* The tear was not directly seen on conventional static utrasound. The thick white arrow identifies the footprint on the humeral head (HUM), and the dotted arrow outlines the humeral cartilage. The dark arrowhead corresponds to the proximal stump, and the light arrowhead to the distal stump of the torn tendon, both delineated by fluid motion within the defect.

• The static evaluation follows standard protocol, with the probe positioned in the coronal and sagittal oblique planes over the greater tuberosity. The tendon is evaluated in terms of its echotexture, thickness, and the presence of hypoechoic tears or defects (Figures 3 and 5).
• During the dynamic phase (Video 1), the patient performs active lateral elevation of the shoulder against the band (Figure 6). The probe is maintained in a stable position (Table 1) in order to facilitate continuous visualization of the tendon. The aim of this dynamic phase is to observe the behaviour of tendons under load, including sliding, gapping, and deformation (Figure 7; Table 2).

**TABLE 1:** Pearls and Pitfalls to Correctly Use the Pilates Band During Ultrasonography

Pearls	Pitfalls
Keep the elbow aligned with the trunk to ensure proper lateral elevation	Forward elbow drift reducing visualization of tendon gapping
Check visible deltoid activation confirming proper elevation	Wrist or elbow substitution mimicking shoulder elevation
Use ample gel and minimal probe pressure	Excessive probe pressure masking subtle defects
Maintain consistent probe orientation during dynamic testing	Changing probe angle affecting reproducibility

**TABLE 2:** Advantages and Disadvantages of the Method

Advantages	Disadvantages
Tendon defect can be seen directly	Requires active patient cooperation
Simple and easy to perform	Limited in painful or restricted shoulders
Low cost	
Nonirradiating	Requires training

## DISCUSSION

The diagnostic performance of US in evaluating rotator cuff tendons is well established.^3^ Nevertheless, obtaining a high‐quality US examination remains challenging. Although tendinous lesions are theoretically hypoechoic, they may occasionally contain hyperechoic or echogenic material (fibrous or hematic tissue), which has the potential to cause diagnostic errors during US interpretation, especially in case of partial full‐thickness.^4^ Furthermore, the operator's experience is key to ensuring accurate diagnostics. Performing and interpreting US of the rotator cuff is an endeavour which requires a considerable degree of training and expertise to be successfully accomplished.^5^ The operator's professional background also significantly impacts the sensitivity and specificity of US in detecting rotator cuff tears; general radiologists without a musculoskeletal focus and nonphysician sonographers showed lower diagnostic performance.^3^, ^6^

Among these advances, dynamic maneuvers and applying resistance during a US examination have proven to be valuable in detecting myotendinous injuries.^7^, ^8^ By applying controlled resistance using a Pilates elastic band during active lateral shoulder elevation, joint fluid mobilization is enhanced, and the tendon is subjected to physiological loading. This tension can unmask fissures through abnormal gapping or altered tendon gliding. This is a simple method that is easily understood by the patient and does not require any particular adjustment to the basic position.

However, it is necessary to ensure that the patient is in the correct position and that the elbow is raised laterally in parallel. A simple way to ensure that the movement is performed correctly is to check for simultaneous contraction of the deltoid fibres in the plane of the probe (Tables 1 and 2). Overall, early and accurate detection of tendon tears is essential for appropriate clinical management. Our preliminary experience suggests that incorporating dynamic resistance US testing into standard protocols enhances diagnostic confidence—particularly among less experienced operators—and may influence treatment strategies, including rehabilitation planning and surgical decision‐making.

## DECLARATION OF GENERATIVE AI

During the preparation of this work, the author(s) used ChatGPT (OpenAI, version GPT‐5) to improve the clarity and grammar of the text. The author(s) reviewed and edited the content as needed and take full responsibility for the content of this publication.

## DISCLOSURES

The authors (V‐T.V., C.V., R.N., N.S., N.B., M.F‐B.) declare that they have no known competing financial interests or personal relationships that could have appeared to influence the work reported in this paper.
